# On the Reform of Lunatic Asylums
*In giving a prominent position to the able and interesting communication with which Dr. Parigot has favoured us, we wish it clearly to be understood that we dissent entirely from the conclusions which the learned Belgian physician has arrived at respecting the *asylum *system of treating the insane. The results of the so-called "*free-air*" system at Gheel, no doubt furnish most valuable hints towards the solution of the important question of the best method of providing additional accommodation for lunatics; and we would refer to a modification of this system, suggested by the Scotch Commissioners in Lunacy in their Second Annual Report, and detailed in a subsequent page of this Journal.—Ed.


**Published:** 1860-07-01

**Authors:** J. Parigot

**Affiliations:** Honorary Professor in the University, and Member of the Committee of Inspectors of Lunatic Asylums of the arrondissement of Brussels, &c.


### THE JOURNAL

OF

### PSYCHOLOGICAL MEDICINE

AND

### MENTAL PATHOLOGY.

JULY 1, 1860.

### ART. I.—ON THE REFORM OF LUNATIC ASYLUMS.*

BY J. PARIGOT, M.D.,

Honorary Professor in the University, and Member of the Committee of Inspectors of Lunatic Asylums of the arrondissement of Brussels, &c.

(*Written expressly for this Journal.*)

" We should like to see the experiment tried in some new district of producing in its integrity the Belgian system."—*Quarterly Review*, 1857.

A WIDE difference of opinion exists amongst alienist physicians of the present day in reference to two essential points in the treatment of the insane. These points relate to the most favourable conditions under which we can place lunatics with a view to their cure, and to the mode in which the public bounty shall in future be applied in providing for the treatment of such patients —supposing it to be necessary that they should all be confined, and that the localities prepared, at great expense, for their reception, are still insufficient in number.

Hence arises a question of reform all the more important because it requires, in several respects, a complete renovation of our ideas on the treatment of insanity. It must be confessed that up to the present time we have been barbarous enough to punish the insane like criminals, to exclude them from society, to place them on a level by law with ferocious and mischievous animals, and, in fine, to abandon them to their fate. A denial of justice such as this, which has been patent for ages, can only be remedied

* In giving a prominent position to the able and interesting communication with which Dr. Parigot has favoured us, we wish it clearly to be understood that we dissent entirely from the conclusions which the learned Belgian physician has arrived at respecting the *asylum* system of treating the insane. The results of the so-called "*free-air*" system at Gheel, no doubt furnish most valuable hints towards the solution of the important question of the best method of providing additional accommodation for lunatics; and we would refer to a modification of this system, suggested by the Scotch Commissioners in Lunacy in their Second Annual Report, and detailed in a subsequent page of this Journal.—ED.

by the civilization *of manners;* let us hope then that our eyes may be opened with reference to this point in the moral history of humanity, at the very earliest possible moment.

It will not suffice to improve our asylums, to attend to their hygiéne, and to render the lot of the insane more bearable; we must cure as quickly as possible the curable (as the Father of Medicine enjoined), and we must not put the incurable to needless pain.

Unhappily the actual state of things gives rise to great difficulties, for at the same time that the growth of a miserable and ignorant population causes mental disorders to increase, it everywhere results that considerable capital is sunk both in public and private speculation for lodging and taking charge of the insane.

But is it necessary that we should delay our search for a remedy, which we know to be needed, until by an excess of misery and the results of our own carelessness, the eyes of the public are opened?

For more than fifty years the countries most advanced in what may be called plastic civilization—that which has taught us to construct and to manufacture, but finds us still desiring somewhat, which on being applied to the science of humanity, shall best develope the mind and the heart—these countries, we say, as well in Europe as America, have founded vast asylums for the insane, sometimes indeed so gigantic that the imagination stands aghast in contemplating them. "What a mass of misery they must contain, and still they are not of sufficient magnitude!" —such are the words that then escape from our lips.

To what a pitch of industrial elevation have we attained, when our most effective mode of succouring the afflicted forcibly leads us to banish them from our sight! There are, indeed, many amongst us who hold that if society sought and desired to direct the education of all to a humanising and religious end, insanity would fare less hardly in the midst of the difficulties of social life; but so long as we are content to seek palliatives in order to render the effects of our neglect less sensible, we must expect difficulties which science can foresee, though it is without the means of combating them with advantage.

In the present day we build asylums everywhere; masonry is the fashion; every province in Belgium, every county in England, and every department in France, it is said, should possess this proof of our solicitude. Physicians of reputation and officials of high rank recommend the measure. This we might expect, for these very honourable men, habituated to magisterial functions, find nothing of greater efficacy to meet every contingency, and the horizon of their ideas appears to be bounded by a desire to possess greater asylums for the *restraint of madness.* But as we already have hospitals for every corporeal infirmity, and as we could not immure all that are afflicted, since the incumbrance would at once imperil the public health and the funds at our disposal, the question regarding the reform of our asylums has naturally become the order of the day, and so will remain until a satisfactory solution be arrived at.

More than thirty years ago physicians of high repute, such as Esquirol, and Moreau of Tours, convinced of the necessity of continuing the reforms commenced before their day, proposed measures, the practicability of which they were led to appreciate, in examining and relating the experience of a Belgian village, in a locality lost, so to speak, in the steppes of Campine, in the province of Antwerp. Since that date, the organization of the colony having been more closely examined, it has been asserted that, by following the example of the Ghéeloise peasants, unhoped for advantages might be attained in the treatment of lunacy, such as the acceleration and augmentation in number of the cures, the diminution of expense, the removal of that species of disgust or reprobation with which insanity is most commonly regarded, and the attainment of a twofold action of benevolence and mutual *moralization* between the patients and those who attend upon them.

If all this be possible, why is the establishment of similar institutions in other countries still delayed?

We will state frankly what are our ideas upon this subject.

In the first place, we may consider as the most perfect asylum models in existence, those establishments where but a small number of patients are admitted, maintained by men of high scientific reputation and undoubted probity. In return for fees proportioned to their reputation and the fortunes of their clients, these physicians offer the most consummate science, the most assiduous care, town and country houses, suitable and sufficient domestic arrangements, and, above all, a *family life*. Accomplishing their duties towards humanity, these distinguished men naturally see no reason to renounce their system. Other physicians, equally to be respected for their science and their zeal, placed at the head of large public or private establishments, which have been well kept and honourably administered, also very naturally defend their domestic hearth, the asylum, which is in some sort their patrimony, since it has very commonly been transmitted to them by their ancestors. Moreover, it would be unreasonable to expect that every one should feel himself equally called upon or disposed to imitate the self-devotion and personal sacrifice of those physicians who strive for reform against their own interests. But, finally, there lives under shelter of the reputation of the conscientious men of whom we have spoken, the mass of speculators and traffickers in madness who, in countries where there are laws for the protection of the insane, only do what is strictly required in order to escape blame, yet who in everything else, or on favourable occasions, only seek to retain captive unfortunate beings whom, most frequently, unnatural relations, from disgraceful motives, wish to get rid of.

It is true that in the abandonment and neglect of the insane we must recognise shades and gradations: the patient is no longer beaten nor injured; he has all that is indispensable, and sometimes the passers-by are imposed upon by the sound of music, dancing, fêtes, and entertainments of various kinds; but if we go to the bottom of these appearances, we often find they proceed from a wish to render the aspect of a prison less disagreeable, while the desire to cure is either altogether absent or but lightly appreciated. In the face of such inveterate evils, it will be easily understood that a reform which is to change the whole face of things will have to suffer delay; but in sounding the evil the conviction forces itself upon the mind that the desired victory will ultimately be achieved.

There are then two chief obstacles to success,—particular interests coalescing against the public good, and a general indifference of public opinion towards the malady, notwithstanding that it so often attacks men distinguished in all branches of intellectual activity, those whose sensibility is too strongly developed, and again those who have fallen victims, from physical or moral causes, to the vices of society,—all of whom deserve our pity.

In the midst of these circumstances opposite opinions have been formed. There are those on the one hand who would reform the public asylums by transforming them into *free-air colonies*, or at least in modifying them so as to unite at once the advantages of an asylum with those of a colony; there are those on the other hand who maintain proposals for purely local amelioration. The system at present in vogue consists in secluding the lunatic in an asylum, where, according to the nature of the case, he is either placed in a barred cell or in one of the divisions of patients; in rare and exceptional cases he is allowed to go out under the guardianship of a keeper. This method, miscalled *curative*, is based upon moral and physical restraint, and is still counselled by very estimable men: the patient is compelled to live in a new world nearly approaching to that of a convent. Such asylums in Catholic countries are very often directed by religious corporations, to whom the undertaking has been entrusted. Assuming then that under this system the mercantile idea is placed in the second rank, and that the requirements of the system are satisfied, still the accumulation of patients is an obstacle in the way of good treatment. But besides this, a contempt of science, and personal interest, insensibly lead to the same result. How can we expect that a physician, to whom is denied pecuniary means and the time necessary to profound study, should maintain the unequal conflict? Again, governors and officials, and even ministers of State, are sometimes of opinion that no reliance is to be placed upon psychical medicine. Amongst all these powerful personages, those who belong to, or are under the influence of, the clergy, think that moral medication is but a dependency of devotion. Were this indeed the case, the religious habit should be the best remedy. Lastly, as a general rule, it is considered *good management* to get any kind of a physician to undertake the care of hundreds of patients at a low remuneration. Such being the system, saving the exceptions which we have referred to, one can understand without difficulty why cures are rare and asylums so well filled.

The reform system proposes on the contrary a complete medical treatment,—that is to say, it embraces all the means, moral and physical, which address our double nature. Its sole object is to return the patient to his family as cured. For this it requires the devoted attention of a competent physician, for the latter will never consider his patients as incurables whom he is at liberty to abandon to nature; he studies and strives for their interest to the last; nothing is indifferent to him. Under this system also all the ordinary circumstances are changed; in order to avoid complications a free space is required, each patient is permitted a degree of personal liberty, and with this view he is placed in the midst of a special society created for him. The attentions generally confided to domestics are replaced by those of a family, whose mission is to render inoffensive a man sometimes furious, and, in consequence, deprived of moral liberty and a knowledge of his acts. It is, so to speak, by a moral *tour de force* before the eyes of those who can only recognise brute matter, that this system commences a medication of the man afflicted, as Lord Byron says, *from on high*.

It will be readily understood that no importance attaches to the name of a system having this end, and which may be adapted to countries of different climates and manners; but as this system is practised in the country and rejects restraint, it has received the name of the *free-air treatment* (*traitement à air libre*). It may be carried out by one or more families in combination, by a village or a colony, without losing its special character. All is to be attained by kindness, not by intimidation or violence. Nothing should be allowed to oppress the individuality of the patient, the spring of intellectual life which, once broken, involves the loss of the individual. The capacity for amelioration and cure by the aid of science is to be taken as inherent in the moral and physical conditions of the patient. No one can be ignorant that the efforts of the man of art must be stronger than the evil which he combats; neither must we forget the labour necessary to trace the origin and seat of evil, to foresee its phases and to determine its treatment; or the time and patience required for these examinations of the patient; nor, on the other hand, the satisfaction of the physician when his labour ends in a happy result.

If we should be asked where this system is completely adopted, we should be compelled to reply that the Ghéeloise Colony approaches the ideal without attaining it; but we hope that, with the concurrence of its present chief physician, the reforms commenced there ten years ago will be continued until they be realized.

Finally, it appears to us that the question of reform is so urgent that the *non-medical* public ought also to be consulted upon the subject. *Res sua agitur;* independently of its direct interest in the matter, the public can very well judge of the practical bearings of the subject, as, for example, which system is the simpler and the less painful; bearing witness at the same time to that which effects the quickest cures.

In the elucidations which we offer in this paper, we shall endeavour to be as impartial as possible; and although it be written in favour of reform, and owes its birth to a controversy raised against us in Germany and in England, we have been moved by a sole regard for the interest of truth.

I. After what has been stated above, it will be easily foreseen that those physicians who are in favour of restraint will contend, that a house in which life is subject every instant to rule and discipline, becomes in some sort an instrument of cure, and they will hasten to add that, according to the celebrated Esquirol, it is the *most powerful therapeutical agent in the hands of an able physician.* It would not be difficult to refute this assertion, so far as it is absolute and general; but it is sufficient to say that it proceeds less from a scientific theory than from an idea of perfection, which is habitually attached to a conventual and religious life. The secular clergy, not restricted to this mode of existence, are from this point of view *irregular:* many men consider, therefore, that monastic life leads to perfection; they think that the passions, which according to them are the sole sources of madness, may be more easily controlled in this species of cloister-life. It may certainly produce very marked effect upon some minds, but we must not forget the effect due to seclusion and removal from the family circle. There is nothing, however, surprising in the fact that a physician such as Esquirol, *well versed in mental therapeutics,* knew how to turn to advantage this moral commotion by instituting a rational treatment, to which most commonly the patient or his family were opposed. But it is not this which, in aid of their cause, the advocates of restraint find it necessary to maintain; taking their stand upon the letter of the aphorism, they pretend that the walls surrounding an asylum possess some mysterious therapeutical action, and they allege, as has been recently stated with reference to an architectural competition for the plan of an asylum near Madrid, that *curative methods** are intimately connected with architectural arrangements, so much that one might more surely arrive at the construction of a good hospital for lunatics by studying the latter rather than in perfecting the former; but this is to maintain that the material distribution of the asylum has some secret relation to maladies of the mind; it is passing from phrenological organology to still greater absurdity,—its imitation in the divisions and subdivisions of quarters, as if there were intellectual madmen, sentimental madmen, and instinctive madmen. Hence proceeds the sophism that the treatment of insanity is capable of assistance from classification. It is impossible; classification serves at best but to render life more endurable to the prisoner, and that is all; the psychopathist worthy of the name rarely finds himself obliged to use as a therapeutic measure the wearisome and painful expedient of four walls.

Thousands of plans have been produced in the search for the *x* of this much desired classification; all the asylums in Europe have been visited and studied by a mass of travelling psychopathists in search of its traces and therapeutic signification, amidst all possible combinations of straight lines and curves; labour in vain, no one has yet discovered the relation between bricks and thought. Psychiatry has nothing to hope for from classification; as a matter of administration it is well to divide the patients into boarders and paupers, turbulents, semi-turbulents, and idiots. But the registers of the administration have no columns reserved for sufferings real or imaginary (the physicians alone know and are able to estimate these), and provided that order reigns, and a physician visits twice a day the halls, warming-places (*chauffoirs*), or workshops, it is considered that all has been done that can be to obtain the cure of the insane.

The time approaches when such exaggerations will be abandoned; it will ultimately be understood that isolation ought not to be confounded with imprisonment; true it is that we must separate the patient from the circumstances which have led to or have witnessed the commencement of his madness, but it is clear as the day that this advantage is obtained as easily by removing the patient as by causing him to be shut up. In the free-air system, the patient adapts himself to the change more easily, he accepts the pretexts invented for it; the head of the family with whom he is placed, treating him well, the salutary effect of isolation is obtained without violence, and above all without risk of exciting a patient who has need of repose.

* *Des articles d'aliénés en Espagne.* Paris. 1859.

The partisans of restraint have, at various times, attempted to make it appear, that personal liberty is both hurtful to the patients and fatal to those by whom they are surrounded, and that the conditions which permit it involve so much peril and danger of abuse, as to render it desirable that the only colony which furnishes the example of it, should disappear as soon as possible.

Happily these insinuations have no foundation. This is apparent, for the least potent of the influences which are looked upon as noxious, having been in action for many centuries, would have destroyed root and branch the very Gheel which we find, on the contrary, to be most flourishing at the present time. The system is good, and what proves this is that, notwithstanding the inherent defects of the colony, as originating from the spontaneous commiseration of ignorant peasants, who themselves are the object of speculations of every kind, this method of receiving patients into their houses for a trifling emolument has been advantageous to all parties. Gheel, moreover, has borne up against certain *ameliorations* which have diminished the number of boarders there. In consequence of a regulation imposed by the State, on the suggestion of a commission, the colony has been deprived of all lunatics suspected, on whatever ground, to have suicidal, homicidal, or dangerous propensities. All the insane coming within these categories have been sent into closed asylums.

The partisans of reform, on the contrary, hold that isolation from the world and its activity is always mischievous, when the excitement of active life does not exceed certain limits. If a restless patient be shut up, all salutary diversion is prevented, and he is subjected to a fatal internal excitation. If he is restrained by a strait-jacket, or bound fast, the unfortunate thus condemned to immobility, undergoes the most atrocious torture; and, finally, shut up with his equals, the constant contact with madness (as we shall subsequently show), adds fresh anguish to his position. Under such circumstances, the phases of the disease most favourable to its cure cannot but pass by rapidly, and the affection quickly degenerates into an irremediable chronic state. This we believe to be one of the most active causes in encumbering asylums.

So far we have stated the arguments of two extreme parties; it is only just to mention the opinion of men who may be considered the eclectics of psychiatry. They lay down as a principle, that the application of any system whatever should depend on the nature of the mental disorders to be treated; thus it is necessary, say they, that we make up our minds to restrain the furious, the melancholics of perverse sentiments, idiots of criminal or scandalous tendency, &c. On the other hand, although they adopt cellular confinement, they permit it only for a very short time; lastly, they agree that restraint, whether mechanical or moral, debases the patient in his own eyes, and they think that recourse should rarely be had to it. The English system of non-restraint probably owes its origin to a repugnance to violent means. Although difficult in its application, it may be said to have succeeded at Hanwell, where more than a thousand patients dwell together in peace; the same system has been adopted at Meerenberg, near Haarlem, in Holland, and is maintained with advantage. At the same time we must not forget that non-restraint is only possible under most paternal control. I would say further that it must often permit evils which it cannot prevent without greater inconvenience: the family is too great; it is necessary in an asylum to consider the mass and to neglect the individual; this is not the case in a private house which the chief can direct without difficulty. Non-restraint in an asylum requires also on the part of the servants and superintendents an uncommon amount of knowledge respecting the characters and actions which are peculiar to madness; it demands very great prudence in order to foresee and avert catastrophes imminent at every instant in such an organization. The government of these asylums is for this reason always somewhat oppressive. Finally, notwithstanding precautions, some victims have expiated their temerity in the midst of lunatics afflicted with a perversion of the will, and detained against their protestations in what they call their prison; indeed it will be allowed that there is a kind of antithesis in giving liberty to a number of lunatics, forcibly kept together within restricted limits.

It appears to us that non-restraint under the old system is the negation of a physical evil, the name indicates this; whilst in a more advanced stage it becomes, as applied in a family, the affirmation of a moral well-being which permits madness to be dealt with like any other malady. The English have made one more step from the *non-restraint* towards the *free-air system*, viz., the *cottage system*, in which the patient is placed in a villa or cottage, dependent upon an asylum, and either isolated in a park or standing in its own enclosure. If I were to define this method, I should say that it is *the free-air system, less the family life and the medical organization* of a colony. In England, criticism has not been wanting when it was seen that this system consisted in the isolation of a patient under one or more keepers, with an accidental visit from a physician; but it is evident that the reform has only one more step to make to establish itself definitely in a country where the practical view of everything is eminently developed.

II. Actuated by a very praiseworthy feeling, Dr. Roller, one of the most distinguished savants in Germany, senior physician to the Illenau asylum, in the Grand Duchy of Baden, lately proposed a modification of the "*free-air*" system. In an article in the *Allgemeine Zeitschrift für Psychiatrie*, and on the occasion of a bibliographic review of a memoir of Gheel, by M. Duval of Paris, inserted in the *Revue des deux Mondes,* he observes that the existence of a free colony, dating several centuries back, contains in itself the proof of its rationality. Thus it is clear that a large number of lunatics, united in a village, have no need of an enclosed asylum; that the patients are more capable of enjoying their liberty than many people think, and that they may live sociably without danger. M. Roller asks if this example ought to be lost. Why not apply it, he says, to the solution of the problem which the increase of population imposes upon public aid? According to M. Roller's plan, the incurables being placed in the neighbourhood of an asylum, would at least allow of the admission of fresh cases; and these last would then be able to receive the attendance necessary to their cure, during the stage when it could be effected. In fact, there exists no sadder evil (England and Germany complain) than the *overcrowding* of an *hospital* or asylum. In this case, the establishment of colonies having become a necessity, let the asylum be central, let it be the therapeutic centre, which every one will approve of; the name matters nothing, and the method proposed is perfectly acceptable.

If M. Roller had paid Gheel a visit, instead of contenting himself with descriptions and reports, perhaps his opinion of the value of this colony would have been different. Thus, this distinguished man thinks there is an opposition between ideas and facts in the words "*liberty*" and "*chains;*" he thinks that the assassination of innocent persons, and the pregnancy of female lunatics, form a sad reverse to the medal, while they do not go to prove the excellence of the principle of liberty for lunatics.

There are some men, the importance of whose opinion is too considerable for us not to seek to rectify it when it is based upon facts imperfectly appreciated; so we think that, on behalf of free colonies and of Gheel, we ought to point out to M. Roller that, if faults have been remarked by ourselves on the subject of the colony, it was with the hope of putting an end to abuses which attach to the best things, and not for the sake of criticising an excellent principle. Can one suppose that circumstances so rare that we might almost pass them over, are to taint a whole population and annihilate the good which it does? Gheel has existed for generations; would it be an exaggeration to suppose that the colony has assembled several hundreds of thousands of invalids? Certainly not. Up to the present time the archives report *two* crimes against the life of the individual; has crime never been committed in a closed asylum?

We are far from hiding the disgust with which the rape of a female lunatic inspires us (whether committed with or without her consent, is of no consequence—it is a crime), but, among four hundred or five hundred women, there are some hysterical patients who manage to elude vigilance even in an enclosed asylum, much more in a colony; and at Gheel, this crime is certainly very rare.

With regard to the irons, chains, and fetters, it is necessary to know what they mean, and then many people would say that the names are more terrible than the things themselves. The question is merely to find a means of preventing a sudden start on the part of restless idiots or maniacs, who might lose themselves in the fields; besides, we are thus rid of the necessity of imprisoning patients; a fetter consists of a small chain, uniting two kinds of bracelets made of iron plates covered with leather; it is attached to the lower part of the leg. We have frequently questioned cured patients who, when at Gheel, had worn these chains, and they have all assured us that it is far better to have one's movements cramped in the fields than to wear a strait-waistcoat in a cell. Besides, when we are willing to pay a keeper sufficiently well to indemnify him for his loss of time, he will take charge of the restless patient, and the chains will disappear.

As to escapes, they are less frequent at Gheel than from enclosed asylums; statistics prove this.

III. There is scarcely any need to insist upon an inspection of the financial side of the question at issue between the asylum *conservatives* and the partisans of reform, for there is a law of economy in psychology which says: "*There is no treatment more expensive than that which does not cure.*"

The public, like government, look for establishments which charge at the lowest possible rate for the keep of lunatics. True, little is paid; but the patient most frequently remains there all his life; where then is the economy? We have calculated, for instance, that for fifty-one years' residence in an asylum an administration had paid more than 14,000 francs for a single lunatic. Notwithstanding the low charge, the sum is considerable; and if we were to inquire into what the average would be for three hundred or four hundred lunatics, the sum would be more considerable still.

The principal question with regard to funds has then for its basis the medical treatment and its ability to perform a cure. Give what is requisite to make treatment useful; recompense suitably the men who are to devote themselves to the cause of humanity; organize a staff sufficient for a certain number of patients; and you will be in a position at the end of the year to judge, by profit and loss, of the service rendered. Of course, as head of this corps, a man must be chosen whose reputation is established; but you must surround him again with young assistants in order that he may leave after him a *school;* this man, eminent in science, must of necessity, as he grows old, slacken his work; and when he is lost, it would be a disaster to science and the country that there should be no one fit to take his place.

We know that the partisans of the old system ask for grand buildings: but at what cost? Millions are fixed in bricks and mortar. If the establishment prospers, it must be enlarged—here are new difficulties. A German alienist physician lately proposed to construct an asylum, the divisions of which separated in the country should form, so to speak, *stations of disease,* through which a patient would successively have to pass before reaching the end of his troubles.

Indeed, the building of palaces "sorrowfully magnificent," as the *Lancet* says, has already cost many millions in Europe. Next comes the *classification,* which requires a repetition of courts, galleries, doors, windows, &c. Each of these objects has given rise to the writing of big volumes for their better construction, in order to effectually shut in the patients; what ingenuity has it not cost to defy mischief, ennui, and the love of liberty! We may ask ourselves now what humanity has derived from all this capital? Has the cost of all these buildings been repaid by cures, or rather are these palaces machines for perpetuating folly?

Clearly, the reform of these abuses would be doubly useful; it would supply hands for work and for produce; in a colony there is little need of keepers, scarcely any of documents, and none whatever of hangers-on; everything should combine without interruption for the care and comfort of the patient; the patient himself even finds employment; every kind of work is open to him, and he repays society by lessening in this way the expense to which he puts it. An infirmary, containing chiefly bathing rooms, rooms devoted to surgery and medicine, and a chapel, and small offices for the use of a whole population, would not probably equal the cost of an ordinary asylum.

But when an asylum has its complement of patients, and a new case arrives, what a fix the managers are in! They are as much embarrassed by this new arrival as if there were no asylum at all. The case is not so in a colony: a colony has no boundaries; it can take in all that offers. When there arrives in a family, a stranger whom they wish to find room for, they inconvenience themselves a little, until a suitable lodging can be got ready. Gheel might take in, without great extra expense, twice its present population.

The expenditure of the principal asylums in Europe has been estimated at from three to five thousand francs a head for each inmate. From this point of view, a colony numbering a thousand lunatics would lead to a saving of 200,000 francs a year, if a village were used in which each cottage should receive from three to four patients, without reckoning the keeper's family. The real advantage which the asylums have over the colonies consists in the possibility of organizing the work in the former; there are all the necessary means for compelling each one to work. All kinds of things are made there; there are trades of all kinds in long workshops, and the various duties of the house are shared by the patients. Now this work has two faults in a therapeutic point of view—first, it is compulsory, and secondly, it is usually not done in the open air. If the work is productive to the rulers of the asylum, the lunatic receives but a small share of the benefit. Hence, right or wrong, the origin of complaints and recriminations prejudicial to every one; *murder* has been known to follow these quarrels.

In a colony, the labour is voluntary, and consequently beneficial. They work who choose, and on conditions regulated by their own fancy. The keeper, his wife, or his children, can induce patients to work better than an overseer, charged with the execution of an unpleasant discipline. Generally, the price of the day's work is higher in places situated in towns; the manager in that case seeks to re-establish the balance according to the work, which is also paid in proportion.

With regard to the rich, they accept work with much difficulty, and hence comes a life of inaction, which does them much harm. Shut up in an asylum, they cost less than if they were in a colony or looked after by a family, but their chance of recovery is also less. Nothing is more sad or more pernicious than to deprive people accustomed to all kinds of diversions, of that liberty which has formed the chief element of their lives. In a colony, the relations and even the distances of social life are preserved. The level of the disease does not reach education or fortune, and as we never lose the innate feeling of ourselves, the moral abasement is not remarked by the inmates. Of this, Gheel presents numerous examples.

However, it is a pity that for persons possessed of a certain competence, and for those who are rich, no one has yet thought seriously of applying the principle of association to found an establishment destined for the cure of sickness in general. How many people, for different reasons, are unable to get themselves suitably treated at home; often they are at a distance from the great centres of population where all kinds of help are abundant; or perhaps economy compels them to apply to men who have not acquired special knowledge, &c. The principle of association which has already solved so many difficulties, steps in here perfectly to answer a general want. A society might offer at a moderate charge all the possible conditions of recovery. Belgium can, from its position, concentrate in a few hours the most eminent science of the countries of France, England, and Germany. Let us suppose that every month general consultations were held; would there be a man rich enough to procure himself such a means of study, discussion, and cure? With regard to insanity, there is no reason why it should not be mingled with other ailments without detection. Three requisites are suggested:—First, country life in a thinly populated district; second, town life near Brussels; third, life at the seaside, of which the pure and bracing air is so great a help. We are convinced that lunatics would find in such an association a means of escaping public notice, and of recovering more speedily than in the most handsome asylum that could ever be designed.

IV. A German alienist physician believed himself called upon lately to set himself up as champion of the asylums which imprison their inmates. In order probably to make himself agreeable to the crowd of speculators we have spoken of, he thought it his duty to set to work to demand the suppression or abolition of Gheel; and why? He knows scarcely anything of the subject, and this want of knowledge is evident in the long article he has edited, and which the *Zeitschrift für Psychiatrie* has been pleased to accept. The best argument he can summon is that Gheel ought to be a practical criticism upon asylums, such as that of which he is the head. Now there would be a very simple plan of clenching the debate upon this point. Let a jury of medical men, lawyers, and philosophers, all impartial men, be empanelled. Let them examine the patients of any asylum named, and compare an even number of those furnished by Gheel *under the most unfavourable* conditions. The jury should decide upon the greatest likelihood of recovery, the good appearance, the air of contentment, and the sum of happiness of each of these patients.

I would wager all I am worth upon Gheel, and for this reason: we know that nervous irritation or excitement among lunatics is principally owing to their often exquisite sensibility, and their almost invariably exaggerated impressionability. Now, by forcing these patients to live together in a kind of inn or cloister, you expose their suffering and delicate natures to innumerable shocks, to insupportable miseries of association, and perpetual dislikes. "I am then truly mad, to be condemned to live with such people as these," cried a despairing monomaniac. To prove what we say, go into any lunatics' sitting-room, and you will be struck with the sight of a fearful assembly of people whose malady consists in their repeatedly recognising themselves to be everywhere and always among maniacs. These men and these women, meeting again and again in different rooms, are dying of ennui; the room in which they pass the night does not belong to them; and this sitting-room, this yard, this garden surrounded by walls, form a kind of cage which they can only leave in an evening to regain their real abode, at least that abode where they experience only their own ennui.

Well! we maintain that their aspect will be the evidence of what they suffer in their minds.

Examine now this other lunatic, who has the enjoyment of free air and the possession of his own room, his own books, his own tools, his own flowers, his own stores, &c., his home is decorated after his own fashion; there are often inscriptions or drawings on the wall which disappear only through the instrumentality of the half-yearly whitewashing. This man is busy in perfecting his dreams: nothing opposes him. He has fields, woods, or extensive heaths at his command; he angles in the rivers or canals; he spreads nets for the birds; in fact, he does with his time just what he pleases. He is most frequently only required to reach the keeper's house by the hours of meals; and if he forgets them, the housekeeper will have his share saved by the common fire. There is another man who all day long traces in the sand of the street the history of his thoughts; they are hieroglyphics of which he alone holds the key. Another finds in walking some mitigation of his nervousness; he is always busy, and returns happy to his dwelling. A score of others go to work with the keeper and his children—the latter are their brothers, their friends; they share the labour of the weakest.

We ask whether, sanitary treatment being the same, Gheel would not carry off the prize? We are certain of it, because, of all human beings, lunatics are those who betray most irresistibly the emotions they undergo.

Among the most determined anti-reformists, we find Dr. Stevens, the resident medical superintendent of St. Luke's Hospital, London. As far as we can recollect, Dr. Stevens, before his visit to Gheel, appeared little prepossessed in favour of colonies, and criticised even the reports made concerning Gheel by a very distinguished English physician. In a paper (which we regret we are not personally acquainted with), inserted in the *Asylum Journal*, and which is quoted by the "Allgemeine Zeitschrift," Dr. Stevens asserts that my honourable successor, Dr. Bulckens, told him, "that he did not possess any means of controlling the exorcisms practised in the chapel of St. Dymphna that if it was in his power to put a stop to them, he should not think it prudent to do so, because what constitutes the colony is not medical science, but faith in St. Dymphna; and that if the saint disappeared, or was neglected, Gheel would have no more cause to exist."

Nothing would so much confirm Dr. Stevens's conclusions as an assent to them by the Medical Inspector of Gheel, the very man whose business is the amelioration of the colony, and who performs his duty with the greatest zeal. Unfortunately, however, Dr. Bulckens affirms, and we have no difficulty in believing him, that he said nothing of the kind. Dr. Stevens, doubtless from want of familiarity with the French language, has evidently misunderstood what was said to him, and even what he saw. At Gheel no one is exorcised; nothing of the kind has taken place for probably a century. Dr. Stevens must have confounded exorcism with the "nine days of devotion" (neuvaine), which is still, though rarely, held in one of the chapels.

Ought not a psychopathist, with the facts before him, to give some better account of what has been termed *blind faith and superstition* in a saint?—and can one think that it is a "relic of barbarism" at Gheel to take good care of the infirm? Do the premises and consequences bear a logical relation to one another?

In our opinion, St. Dymphna bears a character entirely different from other saints who *by right* are of an intolerant nature. The "Sainte Campinaire" is simply charitable; she loves and protects all the wretched who come for shelter to her spiritual domain. *Ego sum charitas* is her motto: hence Jews, Turks, Catholics, or Protestants of all denominations, find a place in the hearts of the Gheelites, her priests. Now-a-days, when everything is bartered for gold, this relic of barbarism is truly extraordinary. To come to the point; the story of St. Dymphna, moral in its essence, handed down from generation to generation,—what might be its origin? There exists no document or legend dating from the time at which she must have lived. Might not the oral tradition of a fact react upon the feeling of a population removed far from any centre of criticism? The idea of helping the unhappy gathered from all sides, has become transformed into the holy image of a young girl resisting the Passions; she is represented in the act of appealing to Heaven on behalf of the wretches who surround her: there is nothing in that to irritate an alienist physician. Neither do we think that Dr. Stevens has any right to suppose that we believe in the mystic virtue of the creed, while he is ready enough to recognise a christian virtue, Charity, hidden beneath the story of the daughter of an Irish king, an obscure individual in a distant era, who attempted the honour of his child.

Now, in what respect can this legend prejudice medical treatment? It is clear that the saint has deserved well of human nature; upon this claim, and upon the ground of respect for conviction, ought the medical man at Gheel to affect contempt for those who wish to have recourse to this reaction of mind over matter? The Saint might be abolished: but what have we to replace her? Psychiatry is quite a new science, and has had to pass through certain stages of development; incarceration, and all the violence undergone by lunatics form one of its most cruel stages, from which we have just escaped. At present we confine ourselves to the study of the *physical* man; *psychical* man is not yet the order of the day, and the proof is that in no University of the State, nor even in any book, is the study of the morbid wanderings of the mind inscribed on the programme of the course. This study, so intimately connected with lesions of the nervous centres, with the troubles of general sensibility, and with nervous diseases, is left to dreamers and psychologists, who, in their character of medical men, are called in England *mad doctors*, and in Germany *zotten doctoren*. No; St. Dymphna is not yet superfluous.

Following an article by Dr. U. Jessen, in which all the arguments against Gheel are crowned by the idea that this colony is a *disgusting* (abschrecken) example of the free-air treatment, we find that Dr. Bucknill, relying upon the observations of Dr. Stevens, compares Gheel to the small English asylums, which he calls with reason, "*squalid asylums*." Apparently the opponents of our opinion have come to hard words: we will not follow them over this ground, but we will ask Dr. Bucknill in what respect the colony can be compared to private houses, where lunatics are maltreated in the grossest manner, while at Gheel what is admired is the devotion and disinterestedness of the keepers.

The *squalid asylums* of England, the expenses of which are scarcely covered by a few rare patients, have this inconvenience, that persons might be detained there unlawfully: at Gheel, the lunatic once cured, there is no power on earth capable of making him stay against his will, and if he wished to live there, the medical officers of the establishment would not allow it. Let Dr. Bucknill come and study Gheel, and we are sure he will no longer say (according to Dr. Jessen's article), that "to create a Gheel is the dream of persons of inexperience, or of weak-minded men."

When colonies are established everywhere, the public will no longer be able to suspect medical men (as has happened), even those whose reputation is best established, of making secret arrangements with managers of private asylums to detain in them, criminally, persons of sound mind.

It appears (still following Dr. Jessen, of Hornheim, near Kiel) that Dr. Brown, inspector of lunatic asylums in Scotland, has made certain observations unfavourable to colonies. As Dr. Jessen reproduces these objections, we will answer the first one, to wit, that the financial administration of a village, subject to feudal rights, would prevent the establishment of a colony, by observing that Government in this country can buy up these feudal rights without injuring any one's interest, but on the contrary to the advancement of every one's interest, if a colony is deemed useful. As to the second objection, that after all the expenses incurred for the keep and clothing of the patients, as in asylums, there would be no profit, we will reply, that this is a great mistake; for admitting even that the expenditure was the same (which is not the case, as we have shown), there would remain a double number of cures to the credit of free air, conjoined with the rejection of useless discomforts for the incurables.

In conclusion, let us remark that all the arguments against free colonies depend upon our interests or our prejudices. The principle which gives rise to the supposition of their adversaries, that they cannot exist, is grounded upon a sophism, namely, that there are no doctors, managers, or keepers, honest and disinterested enough to do good for its own sake. When people are reduced to such objections, they must be indeed near defeat.

In France, several medical men admit the possibility of establishing colonies there. Nevertheless, a very distinguished savant has raised as an objection that the *blackcoats* in a village (taken as a sign of depravity), would be an objection to trusting lunatic females with isolated families: but if there are seven millions of hectares of uncultivated land, and a few hundreds are taken for the colony, it would be like choosing the site of a large establishment: the interest of each family would be the pledge of its morality. Supposing even that the Roman or Latin race were depraved (as is wrongfully asserted), there are in the North and East of France populations of Germanic stock who would suit perfectly.

In Germany and England, the opposition to lunatic colonies is difficult to understand, for they are merely, as in Belgium, the reflection of German thought and Anglo-Saxon common sense.

I conclude by repeating my words before the Society of Medical Science of Brussels, at the convocation of 1st February, 1856:—"I acknowledge, with pain, that the name of Gheel is not appreciated as it deserves to be. But why so? In the first place, you know that the word '*lunatic*' has in it something repulsive; but if it is unfortunate to be insane, it is still more unfortunate, nay it is even dishonourable, according to the notions of the present time, to be *poor*. It is, indeed, the union of the two words, *lunacy* and *poverty*, that causes so many to despise Gheel, whereas, in my estimation, from them arises its glory. Yes, gentlemen, I am convinced that Gheel fulfils a high philanthropic duty, and that its name will become still more illustrious in the records of humanity."

I spoke truly, for since then Gheel has given its name and that of the country to a system which, on account of its humanity, is called "*The Belgian System.*"

---

